# ‘I just thought that it was such an impossible thing’: A qualitative study of barriers and facilitators to discontinuing long‐term use of benzodiazepine receptor agonists using the Theoretical Domains Framework

**DOI:** 10.1111/hex.13392

**Published:** 2021-12-03

**Authors:** Tom Lynch, Cristín Ryan, Cathal A. Cadogan

**Affiliations:** ^1^ School of Pharmacy and Biomolecular Sciences Royal College of Surgeons in Ireland Dubin Ireland; ^2^ School of Pharmacy and Pharmaceutical Sciences Trinity College Dublin Dublin Ireland

**Keywords:** behaviour, benzodiazepines, discontinuation, qualitative, Theoretical Domains Framework, Z‐drugs

## Abstract

**Introduction:**

Existing interventions to reduce long‐term benzodiazepine receptor agonist (BZRA) use lack theoretical underpinning and detailed descriptions. This creates difficulties in understanding how interventions work and how to replicate them in practice. The Theoretical Domains Framework (TDF) can be used to identify behaviour change determinants to target during intervention development.

**Objective:**

To explore barriers and facilitators to discontinuing BZRA use from the perspective of both current and previous long‐term BZRA users.

**Design/Setting and Participants:**

Semistructured TDF‐based interviews were conducted with community‐based individuals with current or previous experience of long‐term BZRA use. Data were recorded, transcribed and analysed using the framework method.

**Results:**

Twenty‐eight individuals were interviewed. Despite commonalities in perceived barriers/facilitators to discontinuing BZRA use within individual TDF domains, individual participants had different experiences of identified determinants of BZRA discontinuation. For example, both similarities and differences existed within and between each participant group in terms of knowledge of the appropriate duration of BZRA use (‘Knowledge’ domain) and experience of withdrawal symptoms (‘Reinforcement’ domain). Compared to previous users, current users typically anticipated more barriers to discontinuing BZRA use and fewer positive consequences of discontinuation.

**Conclusion:**

This study reports on barriers and facilitators to discontinuing BZRA use from the perspectives of current and previous long‐term users. The findings highlight the challenging nature of BZRA discontinuation and a multitude of barriers that impact participants’ behaviour regarding BZRA use. Future work will involve developing a theory‐based intervention to support BZRA discontinuation in primary care.

**Patient Contribution:**

The study included patients as participants.

## 1 INTRODUCTION

Benzodiazepines and Z‐drugs (e.g., zopiclone) are chemically distinct medication classes with similar mechanisms of action involving gamma‐aminobutyric acid, the principal inhibitory neurotransmitter.^1^, ^2^ Benzodiazepines are licensed for several indications, including anxiety and insomnia, whereas Z‐drugs are only licensed for insomnia. Collectively, they are described as benzodiazepine receptor agonists (BZRAs). Guidelines recommend limiting BZRA prescribing to short‐term use (≤4 weeks).^3^, ^4^ However long‐term BZRA use (>3 months) persists worldwide.^5^, ^6^ This is problematic as patients can become physically dependent on BZRAs and experience withdrawal symptoms, even after relatively short‐term use (3–4 weeks).^7^, ^8^ In some cases, patients may continue BZRA use for longer than originally intended to prevent or suppress withdrawal symptoms.^9^ Long‐term BZRA use has several negative consequences, including cognitive impairment and increased risk of falls.^10^ Potential benefits of discontinuing long‐term BZRA use include less daytime sedation, improved cognition, mood and sleep quality and fewer falls.^3^

Interventions targeting long‐term BZRA use have shown varying effects.^11^, ^12^, ^13^, ^14^ For example, cognitive behavioural therapy (CBT) combined with gradual dosage reduction has proven effective in the short term (up to 3 months postintervention) in reducing BZRA use.^12^ However, the precision with which CBT‐based interventions have been described varies and effects have not been sustained over longer periods. Evidence is lacking to support pharmacological interventions (e.g., anticonvulsants, antidepressants) in facilitating discontinuation of long‐term BZRA use.^13^ Brief interventions (e.g., short consultations with healthcare professionals recommending discontinuation) have demonstrated effectiveness in reducing and discontinuing long‐term BZRA use at 6–12 months postintervention.^14^ However, previous interventions have lacked theoretical underpinning and were often poorly described. This makes it difficult to understand how the interventions worked and limits the potential for replication.

In developing interventions, it is increasingly recommended that researchers adopt a systematic approach and explicitly report on the intervention development process.^15^, ^16^ For example, the UK Medical Research Council's framework for developing and evaluating complex interventions advocates using the best available evidence and an appropriate theory‐base to inform intervention development.^17^ Previous qualitative studies have examined patients’ experiences and perceptions of BZRA use.^18^ While these studies have identified various barriers, including dependence, withdrawal symptoms and absence of supports, they have not comprehensively examined both barriers and facilitators or compared and contrasted the experiences of both current and previous long‐term BZRA users.^18^ Furthermore, the studies have not employed a theoretical framework of behaviour change during data collection and many have not specifically included Z‐drug patients.

This study forms part of a larger multiphase project, which aims to develop a theory‐based intervention to support the discontinuation of long‐term BZRA use in primary care. The project aligns with the UK Medical Research Council's complex intervention framework.^17^ The first phase involved systematically reviewing the evidence‐base for brief interventions targeting long‐term BZRA use in primary care.^14^ The second phase seeks to incorporate a theory‐base into the intervention development process by analysing behavioural determinants of discontinuing BZRA use using an integrated framework of behaviour change theories, the Theoretical Domains Framework (TDF).^19^, ^20^

The TDF was developed from 33 psychological theories relevant to behaviour change.^19^ These theories were condensed into domains that are considered mediators (i.e., barriers, facilitators) of behaviour change. The original TDF consists of 12 domains^19^ and a second version (TDFv2), which was produced after a validation exercise, consists of 14 domains.^20^ Both are widely used and researchers are free to choose between them based on familiarity and preference.^21^ The previous systematic review retrospectively coded included interventions using the TDF to identify domains that may have been implicitly targeted.^14^ To ensure that future interventions target relevant behavioural determinants and are theory‐based, discontinuation of long‐term BZRA use needs to be examined in greater detail from patients’ perspectives. This study aimed to explore patients’ views and experiences of long‐term BZRA use. The objectives were to:

1. Identify mediators (i.e., barriers and facilitators) of discontinuing long‐term BZRA use from the perspective of both current and previous users.
2. Select key theoretical domains to target to change the target behaviour (i.e., discontinuation of long‐term BZRA use).

## 2 METHODS

Semistructured interviews were conducted with two patient cohorts using separate topic guides (Supporting Information Appendices S1 and S2) based on TDFv2.^20^ The first cohort consisted of participants that had successfully discontinued long‐term BZRA use. The second cohort comprised participants taking BZRAs on a long‐term basis at the time of interview. The methods were adapted from a previous TDF‐based intervention development study^22^ and the study was reported in accordance with the Consolidated Criteria for Reporting Qualitative studies (COREQ) checklist.^23^ Ethical approval was granted by the Royal College of Surgeons in Ireland (RCSI) Research Ethics Committee (REC1727).

### 2.1 Sampling and recruitment

A multistrand convenience sampling method (outlined below) was used to recruit eligible participants. The following inclusion criteria applied: ≥18 years old, prescribed BZRAs for a period equating to ≥3 months’ supply in the previous year, community‐dwelling in the Republic of Ireland. Patients receiving BZRA prescriptions for any condition (including anxiety and insomnia) were excluded from participation in the following circumstances: cognitive impairment, epilepsy, serious mental illness (e.g., prescribed antipsychotics or lithium) or receiving opioid substitution treatment. This was because the care of these groups and the role of BZRAs within their treatment plans may have been different from individuals who had been prescribed a BZRA for conditions, such as anxiety or insomnia, and remained on the medication for an extended period. Recruitment of both cohorts was conducted concurrently from September 2019 to May 2020.

#### 2.1.1 Community pharmacy strategy

The researcher (T. L.) identified and telephoned community pharmacies in the greater Dublin area (sequenced according to postcode) using a publicly available register, and provided a brief summary of the study to the pharmacist. Pharmacists that expressed interest in facilitating patient recruitment were posted an information pack containing copies of the participant cover letter and information sheet. Pharmacists who agreed to facilitate patient recruitment provided written informed consent.

Recruited pharmacists used an eligibility screening algorithm to assess known patients currently or previously receiving BZRA prescriptions against the above inclusion criteria and provided patients with relevant study documentation (i.e., information sheet, consent form) when they presented in the pharmacy to pick up their regular prescription. Patients interested in participating were asked to provide a contact telephone number to the pharmacist for them to share with the researcher who would then follow‐up with patients directly and complete the recruitment process.

#### 2.1.2 General practice strategy

To supplement the pharmacy recruitment strategy, a convenience sample of three general practitioners (GPs) known to the research team were contacted to seek assistance with recruitment. This followed a similar process to the above pharmacy strategy. The key difference was that eligible patients who received study information were instructed to contact the researcher directly if they were interested in participating. GPs had no further involvement in recruitment and, therefore, were not formally recruited.

#### 2.1.3 Social media strategy

Social media was used to maximize recruitment, particularly in terms of previous long‐term BZRA users who may no longer have been regularly attending a general practice or pharmacy. A brief summary of the study was disseminated through the research team members’ individual Twitter accounts and interested individuals were instructed to make direct contact by telephone or email. The same brief summary of the study information was advertised on an Irish‐based online public message forum (www.boards.ie).

The researcher also emailed podcasts with a specific interest or focus on mental health and/or BZRA use (Supporting Information Appendix S3) seeking assistance in disseminating study information to their listenership together with the researcher's contact details.

### 2.2 Data collection and analysis

Semistructured interviews were conducted in person or by telephone based on patient preference and practical considerations. All interviews were conducted by the researcher (T. L.) between September 2019 and May 2020. All participants provided written informed consent before the interview.

Interview topic guides (Supporting Information Appendices S1 and S2) were based on TDFv2^20^ (Table 1) and piloted on two individuals. Although separate topic guides were designed for each cohort, they followed a similar line of questioning comprising three key areas: (1) information about previous/current BZRA use; (2) perceived barriers and facilitators to discontinuing long‐term BZRA use; (3) interventions/strategies to facilitate discontinuation of long‐term BZRA use.

**Table 1:** Overview of the Theoretical Domains Framework and sample questions used with previous long‐term BZRA users

Domain	Sample questions used with previous long‐term BZRA users
Knowledge	What do you know about reducing or discontinuing long‐term use of these medications?
Skills	Did you have to develop any particular skills to help you in stopping the medication?
Social/professional role and identity	Thinking about the [benzodiazepine/Z‐drug] that you used to take, how did it form part of your identity as a patient?
Emotion	How did you feel when you were first advised to stop taking the medication?
Beliefs about capabilities	How confident were you in your ability to reduce or stop the medication?
Beliefs about consequences	What do you think the benefits of stopping this medication were?
Memory, attention and decision processes	Was there anything in particular that influenced your decision to stop using the mention?
Intentions	Can you remember the specific point in time when you decided that you wanted to try to stop taking the medication?
Goals	How many times did you try to discontinue the medication before being successful?
Reinforcement	Is there anything that would have made it easier for you to stop taking the medication or tried to stop it earlier?
Optimism	How have you coped in managing your symptoms since stopping the medication?
Social influences	Did anyone in particular have an influence on your decision to stop taking the medication?
Environmental context and resources	Did you use any particular resources or supports to help you stop the medication?
Behavioural regulation	Can you think of any practical strategies that have helped you in managing that condition/symptoms instead of this medication?

Abbreviation: BZRA, benzodiazepine receptor agonist.

All interviews were audio‐recorded, transcribed verbatim and deidentified by assigning a unique code. Participants had the opportunity to review and edit their transcripts. Participants were not asked for feedback on the findings.

Interviews within each cohort were first analysed separately, before findings within each data set were compared. Initially, the researcher engaged in a familiarization process by reading and rereading transcripts and listening to audio recordings. The first stage of analysis involved a framework analysis using a deductive approach.^24^ The 14 domains of the TDF were used as predefined coding categories. Interview transcripts were independently coded by the researcher and research supervisor (C. A. C.). Coding was compared and any disagreements were resolved through discussion. Coded transcripts were then uploaded into NVivo QSR 10 software for data management purposes. Data coded to individual theoretical domains within NVivo were then charted into a Microsoft Excel spreadsheet. This document contained indexed summarized data for each participant under each domain, key quotes and references to individual transcripts.

The second stage involved a content analysis of charted data using an inductive approach.^25^ This sought to capture any emerging themes from the charted data. Subthemes were identified and recurrent beliefs expressed by participants regarding each domain were reported. The content analysis was led by the researcher and independently reviewed by the research supervisor. Throughout the analysis, coding was compared and any disagreements were resolved through discussion. Data saturation was assessed by reviewing the charted data under each domain to determine whether similar information was elicited across interviews and if additional interviews were likely to elicit new information.

### 2.3 Identification of key theoretical domains

It was intended at the outset that the selection of key theoretical domains would involve a consensus‐based approach within the research team. The number of times domains were indexed in the framework analysis was to be used as a basic indicator of a domain's particular relevance. The results from the content analysis, such as subthemes and recurrent beliefs, expressed by participants in relation to domains were also to be considered during the process of selecting key theoretical domains to target as part of a future intervention. As noted in Section Design/Setting and Participants, 3 below, this process did not proceed as planned.

## 3 RESULTS

Twenty‐eight participants were recruited comprising 13 previous and 15 current BZRA users. Fifty‐seven percent (*n* = 16) of participants were female and the median age was 46 years (range 24–65). The median duration of BZRA use was 5 years (range 3 months to 21 years). The most common clinical indications for BZRA use were insomnia and anxiety. Each cohort's demographic characteristics were broadly comparable (Table 2).

**Table 2:** Demographic characteristics of interview participants

ID code	Gender	Age (years)	Reason for initial BZRA	Prescribed BZRA(s)	Duration of use
*Previous long‐term BZRA users*					
pBZD_01	Male	59	Muscle spasm	Diazepam	10 years
pBZD_02	Female	43	Insomnia	Zolpidem	3 months
pBZD_03	Male	41	Anxiety	Alprazolam	9 years
pBZD_04	Female	38	Muscle spasm	Diazepam	4 months
pBZD_05	Male	40	Anxiety, insomnia	Diazepam, zopiclone	3.5 and 4 months, respectively
pBZD_06	Male	44	Anxiety, insomnia	Bromazepam, diazepam, zopiclone	20 years
pBZD_07	Female	57	Insomnia	Zolpidem	5 months
pBZD_08	Female	49	Insomnia, panic attacks	Alprazolam, zopiclone	3 months and 1.5 years, respectively
pBZD_09	Female	37	Anxiety	Alprazolam	9 months
pBZD_10	Male	26	Anxiety	Clonazepam	4 years
pBZD_11	Female	58	Insomnia	Zopiclone	15 years
pBZD_12	Female	23	Anxiety, insomnia	Diazepam, zopiclone	4 and 5 years, respectively
pBZD_13	Male	46	Anxiety, insomnia	Alprazolam, zopiclone	3 and 5 years, respectively
Summary statistics	54% (*n* = 7) female	Median age (age range): 42 years (23–59)	Anxiety (62%), insomnia (62%)	Zopiclone (46%), diazepam (38%), alprazolam (31%)	Median duration (range): 4 years (3 months to 20 years)
*Current long‐term BZRA users*					
cBZD_01	Male	44	Insomnia	Zopiclone	17 years
cBZD_02	Female	45	Shoulder injury	Diazepam	8 months
cBZD_03	Male	51	Insomnia	Zolpidem	4 years
cBZD_04	Female	44	Palpitations, insomnia	Alprazolam, zolpidem	1.5 years and 7 months, respectively
cBZD_05	Male	51	Anxiety	Alprazolam	15 years
cBZD_06	Female	62	Anxiety, insomnia	Alprazolam, temazepam	21 years
cBZD_07	Male	45	Anxiety	Bromazepam, clonazepam, zolpidem	20 years
cBZD_08	Male	47	Anxiety	Bromazepam, diazepam	20 and 10 years, respectively
cBZD_09	Male	54	Insomnia	Zopiclone	2.5 years
cBZD_10	Female	65	Anxiety	Alprazolam	1 year
cBZD_11	Female	24	Anxiety	Alprazolam	3 years
cBZD_12	Female	55	Insomnia	Zopiclone	6 years
cBZD_13	Female	29	Anxiety	Diazepam	5 years
cBZD_14	Female	24	Anxiety	Alprazolam	7 years
cBZD_15	Female	39	Anxiety	Diazepam	6 years
Summary statistics	60% (*n* = 9) female	Median age (age range): 45 (24–65) years	Anxiety (60%), insomnia (40%)	Alprazolam (40%), diazepam (27%), zopiclone (27%)	Median duration (range): 6 years (8 months to 20 years)

Abbreviation: BZRA, benzodiazepine receptor agonist.

Most participants were recruited through social media (10 participants through Twitter, 4 participants through www.boardsi.e, 9 participants through the Instagram account of a prominent GP who shared the study tweet as an Instagram story). Two community pharmacies were formally recruited (out of 232 pharmacies contacted) and only two patient participants were subsequently recruited. Three additional participants were recruited after hearing about the study by word of mouth. No participants were recruited through general practices (*n* = 3) or podcasts (*n* = 3). In the latter case, this was because those who contacted the researcher were not residents in Ireland and, therefore, not eligible to participate.

Interview duration varied amongst both participant cohorts (current users: range 25 min to 2 h 16 min; previous users: 32 min to 1 h 46 min). No repeat interviews were required. The multistrand recruitment strategy meant that it was not possible to record details regarding refusals to participation.

### 3.1 Integrated summary of findings

Whilst commonalities existed between participants in terms of perceived barriers and facilitators to discontinuing BZRA use, individual participants had different experiences of the identified determinants of BZRA discontinuation. This precluded a meaningful selection of key domains as the relevance of individual domains varied according to individual participants. Consequently, an integrated summary of findings for both participant cohorts is presented under each theoretical domain below. A summary of the key barriers and facilitators identified within TDF domains (domain name included in brackets) together with illustrative quotes is summarized below. A further overview is presented in Supporting Information Appendices S4 and S5. Compared to the findings from the interviews with previous users, more barriers and fewer facilitators of discontinuing long‐term BZRA use were identified among current users.

Most participants knew that BZRAs were not recommended for long‐term use (‘Knowledge’). All participants knew of potential risks associated with long‐term BZRA use (e.g., dependence, withdrawal symptoms). Several participants in each group were aware of the need for gradual dosage reduction when attempting BZRA discontinuation, which some had learned from their experience of withdrawal symptoms (‘Reinforcement’). Some previous users reported having little initial knowledge of how to safely discontinue BZRA use, and one participant experienced a seizure after abruptly stopping the medication.

I just thought you could stop and you'd be grand, or you'd just come down over a few days and then that would be it, but I ended up having a seizure. (pBZD_06)

Several current users believed their knowledge regarding BZRAs was lacking and some attributed this to receiving a lack of information (‘Environmental context and resources’).

… in the ten years I have been on them, I have never actually been informed or educated on the pros or cons of long‐term benzodiazepine use, ever. (cBZD_14)

Several current users felt they were addicted to BZRAs (‘Social/professional role and identity’).

…I feel that I am addicted to them… (cBZD_13)

Others felt that conditions such as anxiety and insomnia formed part of their identity and necessitated BZRA use. In some cases, BZRA use had either become normalized and part of participants’ daily life, or they could not go anywhere without having the medication with them. Some previous users identified more closely with their underlying medical condition (e.g., insomnia, cancer) as opposed to the medication they were taking.

*…*with the benzos in particular it was a case of I had an illness, I didn't have a need for medication. I had a need to get well. (pBZD_05)

Participants in both cohorts reported mixed views regarding confidence in their ability to discontinue BZRA use (‘Beliefs about capabilities’). Participants across both cohorts that expressed confidence reported either previous experience of stopping the medication or going for a period without it.

Well, last year, I stopped [zopiclone] for a month… and very surprisingly I didn't feel any major withdrawals. I remember thinking to myself, if I ever needed to come off, it wouldn't be difficult (cBZD_01)

Participants who reported lacking confidence had often been negatively impacted by withdrawal symptoms (‘Reinforcement’).

I didn't think I would be able to come off them because the times that I would have had to do without them were horrendous… (pBZD_11)

I wouldn't be too confident at all… I honestly don't think I would be able to get off it because I've tried it, and it's just the brain zaps (cBZD_09)

Across both cohorts, withdrawal symptoms were the most commonly perceived negative consequence of discontinuing BZRA use (‘Beliefs about consequences’). Several participants described negative experiences of withdrawal symptoms (‘Reinforcement’) and negative emotions, such as fear and worry (‘Emotions’), which acted as barriers to discontinuation.

It's a really scary prospect. Not only are you dealing with all the stuff you think is being suppressed but you're talking about dealing with withdrawals as well (pBZD_03)

I'm afraid if I was taken off them what would happen to me and I think it would cause me more stress and worry and I don't know whether the panic attacks would kick in again. (cBZD_06)

Previous users outlined more positive than negative consequences regarding BZRA discontinuation. These included improvements in cognition and reduced daytime sedation. A number of participants across both cohorts believed there were no negative consequences to discontinuing BZRA use. In other cases, participants had either had mixed views or expressed uncertainty towards discontinuation. For example, one current user felt that there was not enough evidence regarding the benefits of discontinuation, and another was concerned that if she stopped BZRA use, she would not be allowed the medication again if she needed it.

Participants described various factors that influenced their decision to discontinue BZRA use (‘Memory, attention and decision processes’). For several previous users, their decision to discontinue BZRA use was linked to their intention not to be reliant on the medication (‘Intentions’). Many of these participants initiated the discontinuation process themselves and, in some cases, without their prescribers’ input. A number of current users also expressed intentions to discontinue BZRA use, some of whom had initiated conversations with their prescribers and three participants were undergoing gradual dosage reduction at the time of the interview. However, they reported similar challenges in achieving discontinuation to other current users. Some participants reported that it took time for these intentions to develop and for them to take relevant actions.

I didn't think it was a long‐term [solution]. I wanted to get back to a place where I had a natural sleep pattern. I didn't think it was a solution to just say, ‘Well, I'm going to stay on [zolpidem] for the rest of my life’. (pBZD_02)

I approached my GP to look at the reduction because I don't particularly like taking [diazepam]. There's quite a lot of side‐effects with it. (cBZD_02)

Few participants in each cohort discussed specific goals relating to BZRA discontinuation (‘Goals’). Some previous users outlined goals relating to minimizing or discontinuing BZRA use.

…after X amount of time [I wanted to] lower it to see how much I could survive on without using a full one. (pBZD_07)

A number of current users were open to BZRA discontinuation, which, for some, was contingent on having a plan for dosage reduction. In a few cases, participants stated they had no intention to discontinue BZRA use as they felt they needed the medication or did not perceive it to have any adverse effects (‘Beliefs about consequences’).

Participants across both cohorts reported varying experiences with different healthcare professionals and in a number of cases participants perceived a lack of input regarding BZRA use (‘Social influences’). Several previous users discussed receiving advice and support from healthcare professionals, including GPs, community pharmacists and psychiatrists, who influenced their decisions to discontinue BZRA use. In contrast, a number of current users reported that their GP either did not support their request to discontinue BZRA use or proactively review and discourage BZRA use. Some current users perceived a lack of insight into BZRA use and methods of discontinuation among healthcare professionals. Across both cohorts, participants’ families and friends had varying degrees of influence on their BZRA use.

The only person who intervened in any way was my pharmacist who expressed concerns that I was taking them for so long (pBZD_13)

I was never advised to stop taking them. I went for the reduction… My GP was quite happy to have me staying on them (cBZD_02)

A number of previous users referred to resources (‘Environmental context and resources’) and practical strategies (‘Behavioural regulation’) that increased their confidence in their ability to discontinue BZRA use. Several previous users had availed of counselling, typically CBT, which they often found beneficial with the dosage reduction process or managing the condition for which BZRAs were originally prescribed. In contrast, fewer current users reported availing of such supports. Participants across both cohorts perceived a lack of available resources for dealing with mental health issues and supporting BZRA discontinuation.

This is a bugbear that I have. I think the whole counselling side of it, the whole support area is dreadful. It is brutally lacking in Ireland (cBZD_07)

A few participants across both cohorts had engaged with online BZRA support groups as a resource for advice and information. Some participants noted the need for caution with their use due to negative content (‘Social influences’).

Sometimes there is a double‐edged sword in those [online] support groups… There's a lot of suffering, so I generally only go there when I need something. If you are to hang out there the whole day you could easily be triggered by a lot of what's happening there… so it's a matter of learning how to use the groups. (cBZD_07)

No current user specifically referred to developing skills relating to BZRA discontinuation (‘Skills’). Previous users made limited reference to the development of additional skills other than coping skills for stressful situations and sleep hygiene skills. Participants across both cohorts reported using fixed dosage reductions at regular intervals over periods ranging from weeks to months (‘Behavioural regulation’). Other participants described taking a fraction of a tablet instead of a full one or limiting the days on which they took the medication with mixed success. A small number of participants in each cohort reported abruptly discontinuing BZRA use with mixed outcomes. Some described this as a negative and unsuccessful experience due to withdrawal symptoms (‘Reinforcement’), while others reported no major withdrawal symptoms.

I just overnight stopped taking the [zopiclone] tablets and I never looked back. No side‐effects. No nothing (pBZD_08)

It was only when they tried to cold turkey me off the drugs was the big failed attempt (cBZD_07)

Some previous users reported that their underlying problem had been resolved or improved through another intervention such as switching to other medications (e.g., antidepressants) during the reduction process (‘Environmental context and resources’). A number of current users had also used additional pharmacotherapies with varying success. In addition to gradually reducing their BZRA intake, participants across both cohorts outlined other strategies and resources they used to deal with the symptoms of the condition for which they had been prescribed BZRAs. In many cases, these supplemented the approach that participants used in attempting BZRA discontinuation. These included exercise, sleep hygiene, mindfulness, meditation, CBT and counselling, breathing techniques and positive self‐talk. For a number of current users, these strategies had mixed success or perceived effectiveness. Some previous users found having a small reserve supply of BZRAs helpful for occasional use if they had several consecutive nights of poor sleep.

I suppose the fact that I do have the [zopiclone] if things get very, very, rough and I am having a really bad night or whatever I can take that and that does help me (pBZD_11)

## 4 DISCUSSION

This study provides a detailed exploration of barriers and facilitators to discontinuing long‐term BZRA use. The study forms part of a systematic approach to developing a theory‐based intervention to support BZRA discontinuation. Previous research in this area has highlighted a lack of transparency in relation to the intervention development process and a lack of theoretical underpinning.^14^, ^26^ Moreover, previous qualitative studies have not employed a theoretical framework of behaviour change during data collection and many have not specifically included Z‐drug patients.^18^ This study advances existing literature on BZRA discontinuation by including the perspectives of two participant cohorts regarding the same target behaviour and incorporating an integrated framework of behaviour change theories (i.e., the TDF). This will support determinants of BZRA discontinuation to be targeted by a future intervention.

The findings highlight the challenging nature of BZRA discontinuation and the range of barriers that impacted participants’ behaviour. The complexity of the target behaviour was compounded by the fact that each participant had their own unique experiences of BZRA use. Differences in participants’ experiences of BZRA discontinuation precluded a meaningful selection of key domains, which is commonly done in qualitative TDF‐based studies,^22^, ^27^ as the relevance of specific domains varied according to individual participants. For example, a number of participants in both cohorts did not know the recommended duration of BZRA use and lacked awareness of appropriate discontinuation methods, with one participant having experienced a seizure as a result of abruptly stopping BZRA use. These knowledge deficits are consistent with previous research.^9^ However, barriers, such as knowledge deficits, did not apply to all participants universally indicating that future interventions may need to be tailored based on individualized assessments of behaviour change mediators.

Withdrawal symptoms are widely recognized as one of the main barriers to BZRA discontinuation. Individuals’ experiences of withdrawal symptoms often vary considerably in duration and severity, which further adds to the challenge of successful BZRA discontinuation.^28^ Withdrawal symptoms (‘Reinforcement’) were a key barrier that affected participants across both cohorts. Interestingly, participants reported experiencing withdrawal symptoms with both drug classes. This highlights the need for support with discontinuation irrespective of the BZRA prescribed.^29^ Several participants reported that withdrawal symptoms led to negative emotions (e.g., fear), which acted as barriers to BZRA discontinuation. The ‘Emotions’ domain appears to have been overlooked in previous interventions targeting BZRA discontinuation.^14^

The findings highlighted marked distinctions between both cohorts in terms of self‐efficacy (‘Beliefs about capabilities’) and outcome expectancies (‘Beliefs about consequences’) of BZRA discontinuation. Compared to previous users, current users typically anticipated more barriers affecting their confidence in their ability to discontinue BZRA use and fewer positive consequences of discontinuation. Although the TDF does not propose testable relationships between domains,^21^ participants’ perceptions within these domains may have had important implications in terms of other relevant domains (e.g., ‘Intentions’, ‘Optimism’), and proceeding with discontinuation. A previous US‐based study found that patients who identified as being dependent on BZRAs felt unable to reduce their use of the medication and reliant on BZRAs to cope with life.^30^ Perceptions of self‐efficacy and outcome expectancies of BZRA discontinuation are important to address as part of any future intervention.

The findings also highlight the important influence of healthcare professionals on participants’ BZRA use and attempts at discontinuation. For example, several participants discussed the positive influence of healthcare professionals (e.g., GPs, community pharmacists) in supporting discontinuation. A proactive approach to promoting BZRA discontinuation is important as patients have reported a passive feeling of trust in prescribers whereby, they expected them to initiate changes in BZRA use if necessary.^18^ However, participants’ experiences with individual healthcare professionals varied. Several participants reported that healthcare professionals either did not support their request to discontinue BZRA use or proactively review and discourage long‐term BZRA use. This reflects the ambivalence that has been reported among prescribers in their attitudes towards promoting BZRA discontinuation.^31^ This needs to be addressed as it could hamper patients from starting or proceeding with BZRA discontinuation. The positive influence of healthcare professionals other than prescribers (e.g., community pharmacists) should be further investigated as this may help alleviate challenges, such as time constraints, that can impede efforts to promote BZRA discontinuation.^32^

Gradual dosage reduction was one of the most commonly reported strategies (‘Behavioural regulation’) used by participants in attempting BZRA discontinuation. This strategy is supported by evidence^33^ and endorsed by multiple guidelines.^3^, ^34^ However, gradual dosage reduction regimens vary in the literature and an optimal tapering schedule has yet to be identified.^35^, ^36^ This was reflected in participants’ reported approaches with some potentially undertaking overly rapid reductions (e.g., taking half a tablet instead of a full one). This could precipitate withdrawal symptoms and hinder success with BZRA discontinuation. Furthermore, a small number of participants in each cohort reported abruptly discontinuing BZRA use, which is not recommended.^4^, ^34^ This further highlights the need to tailor any intervention to support patients in discontinuing BZRA use and ensure any knowledge deficits are addressed.

Another reported strategy that facilitated previous users in proceeding with discontinuation involved having a reserve BZRA supply in case they needed it. Liebrenz et al.^37^ previously proposed maintenance therapy with a long‐acting benzodiazepine in patients with high‐dose dependence due to the challenges in achieving and maintaining abstinence. This proposal has attracted criticism^38^ and evidence to support its use is lacking.^39^ However, in the current study, the rationale for a reserve BZRA supply was not to act as a form of maintenance therapy but for use in exceptional circumstances. This option should be explored further as part of strategies to limit and reduce BZRA prescribing and use.

In addition to gradual dosage reduction, some previous users reported substituting BZRAs for other medications (e.g., antidepressants). However, high‐quality evidence to support using pharmacological interventions in discontinuing BZRA use is lacking.^13^ Most previous users reported engaging with various other resources and supports in discontinuing BZRA use. Several of them had availed of counselling, typically involving CBT, which they often found beneficial. CBT in conjunction with gradual dosage reduction has been found to be effective at reducing BZRA in the short‐term (three‐month follow‐up); however, existing evidence indicates that this effect is not sustained over longer periods.^12^ In contrast, fewer current users reported availing of such supports. One participant who had been referred to a counselling service was still waiting to be contacted by the service. Issues relating to the availability and accessibility of counselling services in Ireland are well recognized.^40^, ^41^, ^42^ This clearly needs to be addressed as part of a holistic approach to reducing long‐term BZRA use and improving mental healthcare. In the absence of adequate resources, previous users may be able to offer important peer support to current users attempting discontinuation.^43^ This needs to be explored further in future research.

This study has also provided useful information regarding recruitment strategies for BZRA‐related research. In anticipation of recruitment challenges, a multistrand sampling approach was used. Participant recruitment via general practices and community pharmacies, which commenced before the COVID‐19 outbreak in Ireland, was largely unsuccessful. It was beyond the study's scope to formally establish reasons for this. However, an unexpected study finding was the vital role of social media in recruiting participants. This may relate to the use of online resources and support groups among individuals experiencing medication‐related withdrawal symptoms.^44^, ^45^ There is some evidence to suggest that social media may be particularly effective for recruiting so‐called ‘hard to reach’ participants through more conventional methods.^46^

### 4.1 Strengths and limitations

The study's strengths include the contextually rich and descriptive data obtained. All interviews were coded independently by two researchers, which enhanced the robustness of the analysis. The analysis of two participant cohorts provides comprehensive insights into behaviour change mediators that can be targeted by a future intervention using behaviour change techniques. This will be outlined in a future paper. In terms of limitations, it must be noted that the results cannot be generalized to the wider population of long‐term BZRA users. Another limitation is that older adults who account for the largest proportion of long‐term BZRA use^5^, ^6^ were not recruited, which reduces the transferability of findings to this cohort. This may have been a consequence of the reliance on social media. While >70% of Irish adults aged ≥50 years have internet access in their own homes, internet use declines with increased age, with social media use experiencing the largest age‐associated decline.^47^ As data collection overlapped with the first national COVID‐19 lockdown in Ireland, it was not feasible to remedy this. Future research should look to develop strategies to optimize recruitment for BZRA‐related research and explore the transferability of findings to older adults.

## 5 CONCLUSION

The study reports on barriers and facilitators to discontinuing BZRAs from the perspectives of current and previous long‐term users. The findings highlight the challenging nature of BZRA discontinuation and a multitude of barriers that exist. As an individual's experiences of determinants of BZRA discontinuation vary, future interventions may need to be tailored based on an individualized assessment of behaviour change mediators. Future work will involve developing a theory‐based intervention to support BZRA discontinuation in primary care.

## CONFLICT OF INTERESTS

The authors declare that there are no conflict of interests.

## Supporting information

Supporting information.

## Data Availability

Research data are not shared due to privacy or ethical restrictions.
