# Supplementary material for: ‘I just thought that it was such an impossible thing’: A qualitative study of barriers and facilitators to discontinuing long‐term use of benzodiazepine receptor agonists using the Theoretical Domains Framework
Source: Health Expect. 2021 Dec 3;25(1):355–65. doi: 10.1111/hex.13392 (PMC8849267; doi:10.1111/hex.13392)
Supplement: Supplementary file 1 — Supporting information. [file HEX-25-355-s001.docx]

# Appendix S1: Topic guide for current long-term BZRA users

Introduction

Thanks very much for making the time to talk to me today.

Have you had a chance to read through the information sheet that was sent out to you?

This is one of a number of interviews that I am conducting with patients like yourself who have been using benzodiazepine/Z-drug medications on a long-term basis. The aim of this interview is to explore your views and experiences of using these medications. I should emphasise from the start that I am not here to advise you to make any changes to any of the medicines that you are currently taking. By asking you about your own experiences of using these medications, I am hoping to get an insight into things that might be important for researchers like me to consider in developing an intervention or strategy to help patients who might want to reduce or stop their use of these medications with the support from healthcare professionals.

Do you have any questions before we get started?

I might start by asking how long have you been taking [benzodiazepine/Z-drug]?

And for what reason were you initially prescribed [benzodiazepine/Z-drug]?

Has anyone ever recommended any changes to your current [benzodiazepine/Z-drug] prescription?

## Knowledge

**Q.** Could you tell me what you know about the [benzodiazepine/Z-drug] that you are taking?

- What is it used for?
- How long should it be used for?

**Q.** And what about discontinuing benzodiazepines/Z-drugs? What do you know about reducing or discontinuing long-term use of these medications?

## Skills

**Q.** If you wanted to attempt to reduce or stop using this medication, is that something you feel that you would be able to do?

Follow-up: How would you go about it?

Follow-up: What would help you to do that?

## Social/professional role and identity

As I mentioned before, I am interviewing a number of patients as part of this study, some of whom have other health conditions and take specific medications for these conditions.

For example, some diabetic patients might take insulin and some patients with heart disease might take blood pressure medication.

Thinking about the [benzodiazepine/Z-drug] that you are taking, how does it form part of your identity as a patient?

Follow-up: What role does it play in your day to day life?

## Emotion

**Q.** How would you feel if you were advised to stop taking the benzodiazepine/Z-drug that you are currently taking?

## Beliefs about capabilities

**Q.** How confident would you be in your current ability to reduce or stop the [benzodiazepine/Z-drug] that you are taking?

**Prompt:** Was there ever a time when you felt more confident than you do now?

## Beliefs about consequences

**Q.** What do you think the benefits of discontinuing this medication are?

**Q.** Can you think of any downsides of discontinuing this medication?

Memory, attention and decision processes

Weighing up these benefits and negatives, how would that influence your decision on whether or not to stop the long-term use of the [benzodiazepine/Z-drug]

## Intentions

**Q.** Have you ever considered, or thought about, reducing or stopping your use of this medication?

**Prompt:** Going forward, is it something that you would consider?

## Goals

And following on from my previous question, have you ever previously tried to reduce or stop using this medication?

How did you go about it?

## Reinforcement

What would have to happen for you to reduce or stop using the [benzodiazepine/Z-drug] that you are currently taking?

## Optimism

How would you cope in managing your symptoms without the medication?

## Social Influences

**Q.** Would anyone have an influence on your decisions about stopping this medication?

**Prompts:** partner/spouse, friends, family, doctors, pharmacists, support groups, community gatherings, clubs?

**Prompt:** Could you tell me more about how this happens?

## Environmental Context and Resources

[Give patient an example of a resource such as EMPOWER educational resource]

**Q.** What resources or supports might help you to stop or get ready to stop benzodiazepines/Z-drugs?

**Prompt:** Do you have access to counselling /support groups?

## Behavioural regulation

You mentioned at the start of the interview that you were taking the medication for [refer to original response], can you think of any practical strategies that might help you in managing that condition/symptoms instead of this medication?

Previous interventions

Previous research has shown that there are a number of approaches that can be used to help patients in reducing and even stopping their long-term use of these medications. One of these approaches is known as a brief intervention – this involves a short consultation with a healthcare professional or written information that advises patients to consider gradually reducing their use of the medication and offers some guidance on how to do this.

If an intervention or strategy was to be put in place, what would you like to see it involve?

- What information would it need to contain?
- How would you like to see delivered?
  - **Prompt:** face to face, written, electronic
- What healthcare professionals would you like to see involved in delivering it?

Finally, if we were to test this intervention in a future study we would need to identify outcomes to measure and see if the intervention had worked.

What outcomes do you think would be important to include in any future study?

That brings us to the end of the interview. Do you have any additional comments relating to anything we have discussed that you would like to make?

Thank you again for speaking with me today

**End of interview**

# Appendix S2: Topic guide for previous long-term BZRA users

Introduction

Thanks very much for making the time to talk to me today.

Have you had a chance to read through the information sheet that was sent out to you?

The aim of this interview is to explore your views on long-term benzodiazepine/Z-drug use. This is one of a number of interviews that I am conducting with patients like yourself who have previously used these medications on a long-term basis. I should emphasise from the start that I am not here to advise you to make any changes to any of the medicines that you are currently taking. By asking you about your own experiences of using these medications, I am hoping to get an insight into things that might be important for researchers like me to consider in developing an intervention or strategy to help patients who might want to reduce or stop their use of these medications with the support from healthcare professionals.

Do you have any questions before we get started? I might start by asking how long have you been taking [benzodiazepine/Z-drug]?

And for what reason were you initially prescribed [benzodiazepine/Z-drug]?

Has anyone ever recommended any changes to your current [benzodiazepine/Z-drug] prescription?

## Knowledge

**Q.** Could you tell me what you know about the [benzodiazepine/Z-drug] that you previously took?

- What was it used for?
- How long should it have been used for?

**Q.** And with regards to discontinuing benzodiazepines/Z-drugs? What do you know about reducing or stopping long-term use of these medications?

## Skills

**Q.** How did you go about reducing/stopping benzodiazepines/Z-drugs?

**Prompt:** Did you have to develop any particular skills to help you in stopping the medication?

## Social/professional role and identity

As I mentioned before, I am interviewing a number of patients as part of this study, some of whom have other health conditions and take specific medications for these conditions.

For example, some diabetic patients might take insulin and some patients with heart disease might take blood pressure medication.

Thinking about the [benzodiazepine/Z-drug] that you used to take, how did it form part of your identity as a patient?

Follow-up: What role did it play in your life?

Follow-up: How has your life changed since stopping the medication?

## Emotion

**Q.** How did you feel when you were first advised to stop taking the medication?

**Follow-up:** How do you feel now having stopped the medication?

## Beliefs about capabilities

**Q.** Thinking back to when you were first advised to stop the medication, how confident were you in your ability to reduce or stop the [benzodiazepine/Z-drug]?

## Beliefs about consequences

**Q.** What do you think the benefits of stopping this medication were?

**Q.** Were there any downsides to stopping this medication?

Memory, attention and decision processes

Was there anything in particular that influenced your decision to stop using the [benzodiazepine/Z-drug]

## Intentions

**Q.** Can you remember the specific point in time when you decided that you wanted to try to stop taking the medication?

What had changed since you first started taking the medication?

## Goals

**Q.** How many times did you try to discontinue [benzodiazepine/Z-drugs] before being successful?

**Follow-up:** How did you go about these attempts?

## Social Influences

**Q.** Did anyone in particular have an influence on your decision to stop taking the medication?

**Prompts:** partner/spouse, friends, family, doctors, pharmacists, support groups, community gatherings, clubs?

**Prompt:** Could you tell me more about how this happens?

## Reinforcement

**Q.** Is there anything that would have made it easier for you to stop taking the medication or tried to stop it earlier?

## Optimism

How have you coped in managing your symptoms since stopping the medication?

## Environmental Context and Resources

**Q.** Did you use any particular resources or supports to help you stop the medication?

**Prompt:** Did you have access to counselling/CBT/support groups?

## Behavioural regulation

You mentioned at the start of the interview that you were taking the medication for [refer to original response], can you think of any practical strategies that have helped you in managing that condition/symptoms instead of this medication?

Is there any advice that you would have for someone who is currently taking the medication and wants to try to stop?

And what about the people who have been advised to stop but choose not to follow the advice?

Previous interventions

Previous research has shown that there are a number of approaches that can be used to help patients in reducing and even stopping their long-term use of these medications. One of these approaches is known as a brief intervention – this involves a short consultation with a healthcare professional or written information that advises patients to consider gradually reducing their use of the medication and offers some guidance on how to do this.

If an intervention or strategy was to be put in place, what would you like to see it involve?

- What information would it need to contain?
- How would you like to see delivered?
  - **Prompt:** face to face, written, electronic
- What healthcare professionals would you like to see involved in delivering it?

Finally, if we were to test this intervention in a future study we would need to identify outcomes to measure and see if the intervention had worked.

What outcomes do you think would be important to include in any future study?

That brings us to the end of the interview. Do you have any additional comments relating to anything we have discussed that you would like to make?

Thank you again for speaking with me today

**End of interview**

# Appendix S3: Podcasts used to disseminate study information

Sigma (<https://sigmanutrition.com/>),

Benzodiazepine Awareness with Geraldine Burns (<https://podcasts.apple.com/us/podcast/benzodiazepine-awareness-with-geraldine-burns/id1358022441>)

The Benzo Free Podcast (<https://www.benzofree.org/features/benzofreepodcast/>)

# Appendix S4: Overview of current users’ perceived barriers and facilitators to discontinuing long-term BZRA use

| **Current long-term BZRA users** | | | |
| --- | --- | --- | --- |
| **Domain** | **Barriers** | **Facilitators** | **Illustrative quotes** |
| Knowledge | - Lack of knowledge of recommended duration of use - Lack of knowledge of potential risks associated with long-term BZRA use^#^ - Lack of knowledge of how to safely discontinue BZRA use | - Knowledge of recommended duration of use - Knowledge of potential risks associated with long-term BZRA use - Knowledge of how to safely discontinue BZRA use | *“I know definitely short term but in my head I am going, ‘No longer than three months’, but even that sounds a bit long”* **cBZD_15**  *“So I suppose I haven't tried to stop them myself but I'm aware of these whole psychological and physical issues with discontinuing them. That physically you can have withdrawal effects and feel horrible trying to come off them depending on dose etc”* **cBZD_04**  *“So I don’t really know the full extent of the repercussions of taking them.”* **cBZD_13**  *“I have no information on [reducing/discontinuing long-term BZRA use] because the information I was given was I need to sleep and that I would be using this long term. I resign myself to using this long term.”* **cBZD_10** |
| Skills | - None identified | - None identified | None |
| Social/professional role and identity | - Underlying condition forming part of individuals’ identity and necessitating BZRA use^#^ - BZRA use forming part of individuals’ identity/daily life - Identifying as being addicted to or dependent on BZRAs^#^ | - None identified | “*Oh my god, it takes over my life, massively, yeah. I suppose if you were to say, ‘how many good days out of a seven-day week’, you would probably only get two with five filled with anxious thoughts and anxiety*” **cBZD_12**  *“It has become part of walking and talking. I have done it for so long, it is part of me.”* **cBZD_01**  *“I feel that I am addicted to them…”* **cBZD_13** |
| Beliefs about capabilities | - Lack of confidence in ability to discontinue BZRA use - Negative impact of withdrawal symptoms on confidence in ability to discontinue BZRA use^#^ - Negative impact of fluctuating symptoms or changes in personal circumstances on confidence in ability to discontinue BZRA use^#^ | - Confidence in ability to discontinue BZRA use - Confidence from previous experience of stopping BZRA use or going for a period without the medication | *“…I know if my [alprazolam] was decreased, with the intention of taking me off it, I wouldn’t be able to. I just wouldn’t be able to.”* **cBZD_05**  *“But right now I can say that I would be confident, I feel like, I know how to manage my anxiety. But at certain times I don’t know if I would be able- but for the most part yeah, I would be confident enough, but it is knowing how to manage it”* **cBZD_15**  *“Well, last year, I stopped it for a month… and very surprisingly I didn't feel any major withdrawals. I remember thinking to myself, if I ever needed to come off, it wouldn’t be difficult”* **cBZD_01** |
| Optimism | - Negative outlook towards BZRA discontinuation^#^ | - Optimistic outlook towards BZRA discontinuation | *“Probably pretty negative and I have a poor impression of [discontinuing BZRA use]. I know that from my own personal experience with my mother”* **cBZD_14**  *“I'd like to think I will cope well, but I'm prepared for the fact that I might not cope due to psychological dependence, if that makes sense”* **cBZD_02** |
| Beliefs about consequences | - Potential for withdrawal symptoms - Potential for re-emergence of original symptoms^#^ - Uncertainty regarding positive consequences of discontinuing BZRA use - Lack of continued availability of BZRAs if needed | - Positive consequences of discontinuing BZRA use (e.g. increased alertness, reduced day time sedation) - No perceived negative consequences to discontinuing BZRA use | *“I am afraid of if I was taken off them what would happen to me and I think it would cause me more stress and more worry and I don’t know whether the panic attacks would kick back in again”* **cBZD_05**  *“I want to see medical evidence and to talk more about the benefits [of stopping BZRA use]… I don’t see clearly enough a long list of benefits, all I see is a list of cons”* **cBZD_14**  *“The only downside is that I won’t be allowed to go back on anything again, so my doctor said I won’t be able to go back on any other benzodiazepine ever again……. so I would be afraid, if something ever came up again”* **cBZD_16**  *“I will probably feel a bit more alert during the day”* **cBZD_01** |
| Reinforcement | - Experience of withdrawal symptoms - Dealing with the symptoms for which BZRAs initially prescribed | - Absence/lack of withdrawal symptoms | *“Yeah, I tried to [taper] myself but the side effects for me were awful…”* **cBZD_02**  *“Well, last year, I stopped it for a month. Actually, I stopped all my tablets for a month and, very surprisingly, I didn't feel any major withdrawals.”* **cBZD_01** |
| Intentions | - Lack of intention to discontinue BZRA use^#^ - Perceived continued need for BZRA use^#^ | - Intention to discontinue BZRA use | *“If I thought that [alprazolam] was having an adverse effect on me, then I would get off them tomorrow. Without a doubt. But for me, it doesn’t have an adverse effect. So the idea of even contemplating on reducing them, with the intention to giving them up would certainly be a non-runner for me.”* **cBZD_05**  *“I want to have it… it keeps me relaxed.”* **cBZD_11**  *“Long term, I’m definitely going to be off it. I don't really particularly like taking them”* **cBZD_01** |
| Goals | - None identified | - Goal of reducing/discontinuing BZRA use - Goal of no longer being dependent on BZRAs^#^ | *“I would say definitely within a year, I would like to be off it.”* **cBZD_01**  “*I will be off them by Christmas. I've actually purposely booked a week’s annual leave, the first week of January and I'm determined that I will be finished them when I go back to work after that.”* **cBZD_02** |
| Memory, attention and decision processes | - Weighing up pros and cons of discontinuation^#^ | - Comparable symptomatic relief from any alternative intervention or non-BZRA treatment^#^ - Option of having continued access to BZRAs if needed^#^ - External influences (e.g. healthcare professionals) prompting reconsideration of BZRA use - Experience of harm from BZRA use^#^ | *“It’s such a hard one - I think that I am at the stage where I am very equal on both sides”* **cBZD_15**  *“…if you were to tell me today that I could take something else that didn’t have the side-effects, that would give me the same result in terms of easing my anxiety and calming me down, I would obviously choose that”* **cBZD_11**  “*Well I often thought about doing this trial where I stop taking it altogether or reduce it down … I feel that I would like to have the option, at any time, if I did give up, that I could take one as required. So if I stopped it, that I could go down to the GP and say ‘Listen, I haven’t slept good for the last few weeks’ and she would give me a week’s supply or the two weeks supply”* **cBZD_01**  *“I would take something else, if you said that would be better. And if you could guarantee me that I would have the same reaction”* **cBZD_10** |
| Environmental context and resource | - Lack of provision of relevant information regarding BZRA use - Pharmacological substitution (e.g. antihistamines)^#^ - Availability of non-BZRA alternatives^#^ - Commitment required with non-BZRA alternatives^#^ - Access to resources and supports for discontinuing BZRA use - Repeat BZRA prescribing system - Lack of support from healthcare professionals regarding discontinuation of BZRA use - Negative information regarding BZRA discontinuation process posted on online fora | - Support from healthcare professionals regarding discontinuation of BZRA use - Pharmacological support to augment dosage reduction process (e.g. antidepressants) - Access to resources and supports for discontinuing BZRA use | *“I was never told anything about that, or withdrawal or about any of these drugs. I was told they were all safe….”* **cBZD_07**  *“I was in my chemist one day and I was getting them and the chemist girl said ‘Oh we have this anti-histamine like an over the counter one’ and I took it and oh my god it was horrendous I was like hallucinating… it was just the worst thing ever”* **cBZD_03**  *“My GP said that he will send me to the counselling…because, as I told you, I have anxiety but I am still waiting [for them] to call me…”* **cBZD_11**  *“I think the commitment you have to make to therapies that are non-medication related is so much harder than committing to, like even if you realise the pros and cons and the side effects and things like that…”* **cBZD_14**  *“I suppose that I am seeing someone and it is through a work scheme and I wouldn’t have the funding to go and see someone on a regular basis…”* **cBZD_14** |
| Social influences | - Lack of input from healthcare professionals regarding BZRA use/discontinuation - Variation in experience regarding BZRA use/discontinuation with different healthcare professionals^#^ - Perceived lack of insight into BZRA use and discontinuation methods among GPs^#^ - Encouragement from family members to continue BZRA use^#^ - Negative nature of material posted online by online forum users regarding BZRA discontinuation^#^ | - Advice and support from healthcare professionals regarding discontinuation of BZRA use - Support from family and friends regarding discontinuation of BZRA use | *“I was never advised to stop taking them. I went for the reduction... My GP was quite happy to have me staying on them”* **cBZD_02**  *“What I found with psychiatrists and GPs [is that] they had no idea of how difficult it is to come off these drugs. They grossly underestimate [it].”* **cBZD_07**  *“I think when I went to the GP I was like, ‘I want to come off them, I was talking to the pharmacist and she said I shouldn't have been on’ them and then she kind of went through it and said ‘you’ve never been reviewed?’ and I said ‘no’. And then I said to her [that] the pharmacist had said short-term intervention.”* **cBZD_02**  *“Sometimes there is a double-edged sword in those support groups. I will be brutally honest with you, there is a lot of suffering - so I generally only go there when I need something. If you are to hang out there the whole day you could easily be triggered by a lot of what’s happening there. There is an awful lot of pain in a bad way, so it’s a matter of learning how to use the groups.”* **cBZD_07** |
| Emotions | - Negative emotions (e.g. fear, worry) regarding discontinuation of BZRA use - Feeling of uncertainty regarding consequences of stopping BZRA use - Fear of being without access to BZRA medication^#^ | None identified | *“…if my [alprazolam] were to begin to reduce, with the intention of taking them off me, that would be a major, major worry to me because number one, they work as a security blanket in the psychological sense, knowing that I have them there.”* **cBZD_05**  *“…it isn't the dependency of the drug that I'm frightened of, I'd be fearful of the fact that my sleep- I'm worried about my sleep. What will happen to my sleep?”* **cBZD_10** |
| Behavioural regulation | - Abrupt discontinuation/Overly rapid dosage reduction^#^ - Tablet shaving^#^ - Limiting BZRA intake (i.e. using sparingly or omitting doses) ^#^ - Lack of availability of supports throughout gradual reduction process^#^ - Effectiveness of alternatives to BZRAs (e.g. herbal remedies, breath techniques, counselling) ^#^ | - Use of gradual dosage reduction - Limiting BZRA intake (i.e. using sparingly or omitting doses) ^#^ - Use of resources and supports to augment dosage reduction process (e.g. sleep hygiene, mindfulness, counselling) | *“It was only when they tried to cold turkey me off the drugs was the big failed attempt”* **cBZD_07**  *“…[somebody] that hasn’t got a clue told me to shave a little bit off myself, off the tablet- and that my body wouldn’t notice it- with a scalpel, take a tiny, tiny, bit away…, and I noticed it, I really, it really affected me and I was barely taking anything off it…”* **cBZD_16**  *“I’ve tried the herbal teas… I’ve tried all them and nothing gives me… nothings worked”* **cBZD_03**  *“I tried that where they teach you how to breath, and all that there CBT and I actually got a panic attack and there’s nothing I can do except take [diazepam].”* **cBZD_09**  *“I’m doing 5% cuts every three weeks and I try to be as specific as possible with the measurements.”* **cBZD_07**  *“I find mindfulness, like a huge kind of help but sometimes or I suppose when you have an anxiety attack or sometimes you are so far into it that the mindfulness doesn’t help, you know it is only in some scenarios and it is great when it works”* **cBZD_12** |

^#^ Denotes barriers and facilitators that were unique to each patient cohort

# Appendix S5: Overview of previous users’ perceived barriers and facilitators to discontinuing long-term BZRA use

| **Previous long-term BZRA users** | | | |
| --- | --- | --- | --- |
| **Domain** | **Barriers** | **Facilitators** | **Illustrative quotes** |
| Knowledge | - Lack of knowledge regarding recommended duration of use - Lack of knowledge of how to safely discontinue BZRA use | - Knowledge of recommended duration of use - Knowledge of potential risks associated with long-term BZRA use - Knowledge of how to safely discontinue BZRA use | *“I know now they’re recommended for short-term use only.”* **pBZD_05**  *“I knew that for long-term use I wouldn’t be able to stay on it because it would become addictive and again you would need more and more...”* **pBZD_07**  *“I just thought you could stop and you’d be grand or you’d just come down over a few days and then that would be it, but I ended up having a seizure because of it.”* **pBZD_06**  *"I think I had the right amount of advice. I wasn't lacking any information or guidance. I'd have to say I was told what the effects would be of not taking them and they were spot on."* **pBZD_01** |
| Skills | None identified | - Ability to undergo gradual dosage reduction^#^ - Development of skills to manage the condition for which BZRAs were initially prescribed^#^ | *“...I've learned measures, like sleep hygiene measures and mindfulness, to try and improve it”* **pBZD_02**  *“I developed what people would call coping mechanisms for social situations - for getting on aeroplanes, for being in the company of certain people - that I can use now that I don’t necessarily need medication to get me through those kind of things.”* **pBZD_09** |
| Social/professional role and identity | - BZRA use forming part of individuals’ identity/daily life | None identified | *“To be honest it became my identity like for me I was so co-dependent it would have been for me like a disease”* **pBZD_12** |
| Beliefs about capabilities | - Lack of confidence in ability to discontinue BZRA use | - Confidence in ability to discontinue BZRA use - Confidence from previous experience of stopping BZRA use or going for a period without the medication - Confidence from practical strategies and resources that facilitated BZRA discontinuation | *“Not [confident] at all... I wasn’t really determined at the time. I didn’t see the benefits of it at the time. So, no, I wasn't confident about it at the time.”* **pBZD_03**  *“I was very confident because I am very stubborn and it was a case of, even starting it I knew it was a crutch and I didn't want to rely on it long term but let myself get into that habit of using it”* **pBZD_05**  *“I was fairly confident because it wasn’t the case that I needed them every day. I just needed them as needed. So, it certainly wasn’t an issue to just stop.”* **pBZD_07** |
| Optimism | - Pessimistic nature of online material regarding BZRA discontinuation^#^ | - Optimistic outlook towards BZRA discontinuation | *"When I was giving up the zopiclone, I really still wasn’t sure that I could and I remember looking up forums and looking up videos of people that had given them up and honestly there was nothing positive like it scared me so much… so I found that really hard… and I just thought that it was such an impossible thing”* **pBZD_12**  *"… it’s not gonna be as bad as you think without being prescribed more drugs, you know."* **pBZD_06** |
| Beliefs about consequences | - Potential for withdrawal symptoms - Lack of perceived positive consequences for discontinuing BZRA use | - Positive consequences of discontinuing BZRA use (e.g. improved cognition, reduced day time sedation, improved work performance) - No perceived negative consequences to discontinuing BZRA use | “*And I am finding that my brain feels more sharp and intelligent*” **pBZD_04**  *“My interest in my work and the quality of my output improved hugely when I came off them. The actual volume of output improved. If I was to look at the work done, for the three months that I was on benzos it would probably equate to three weeks or one.”* pBZD_05 *“…and just the liberating feeling of being able to go out and not been conscious of that tray in my back pocket*” **pBZD_03**  “*No there absolutely isn’t any downsides to stopping it. No.”* **pBZD_11** |
| Reinforcement | - Experience of withdrawal symptoms - Dealing with the symptoms for which BZRAs initially prescribed | - Absence/lack of withdrawal symptoms - Experience of positive consequences from discontinuing BZRA use^#^ - General sense of achievement from discontinuing BZRA use^#^ | *“I remember very clearly going through withdrawals with them thinking I was going to die. It was like [the film] Trainspotting or something like that...”* **pBZD_04**  *“I did try and give them up cold turkey once, off my own choice… it was for about eight weeks that I gave them up, so they were probably the worst eight weeks of my life… So that was the first time and then I went back on them again”* **pBZD_12**  *“… because I came off them over a couple of weeks and because I had that safety net, no, I didn't have any withdrawal or side effects when I came off either”* **pBZD_02**  *“I was kinda more prepared for the process of this for the zopiclone but I did find the zopiclone much, much harder to come off than the diazepam….”* **pBZD_12**  *“Stopping initially was tough... All of the anxious symptoms came back with a vengeance. They felt stronger than before.”* **pBZD_03** |
| Intentions | - Time for intentions to develop regarding BZRA discontinuation^#^ | - Intention to discontinue BZRA use - Intention not to be reliant on BZRAs^#^ | *“…it was a slow realisation over an extended period. It built up momentum and then I said, ’let’s do this’. I didn’t just stop instantly I knew enough about it to know that that’s not a good strategy and to wean yourself off is the best way and so I started to do that”* **pBZD_13**  *"Well I was contemplating it before [the seizure], that you shouldn’t be taking them for so long, yeah, but you’re kinda stuck in a cycle, do you get me?"* **pBZD_06**  *“I suppose I didn't think it was a long term [solution]. I wanted to be able to get back to a place where I had a natural sleep pattern. I didn't think it was a solution to just say, ‘Well, I'm going to stay on [zolpidem] for the rest of my life’.”* **pBZD_02**  *“So I know that you can get into a situation where you do become very dependent on them, and so that wasn’t something that I was going to do, or the doctor was going to do, so I would have been reluctant to stay on them long-term”* **pBZD_07** |
| Goals | None identified | - Goal of reducing/discontinuing BZRA use | *“…after X amount of time [I wanted to] lower it to see how much I could survive on without using a full one.”* **pBZD_07**  *“Every time I saw my psychologist I would remind her that the aim is to get me off these tablets if we don’t achieve this I will consider this whole process not to be a failure but that we didn’t achieve the main aim you know”* **pBZD_06** |
| Memory, attention and decision processes | None identified | - Improvement/resolution of underlying medical problem^#^ - External influences (e.g. healthcare professionals, family, friends) prompting reconsideration of BZRA use - Significant life events prompting discontinuation of BZRA use^#^ - Option of having continued access to BZRAs if needed | *“….it just happened organically that whatever the [escitalopram] I was on meant that I didn’t need them as much as I needed them before. And they were really initially only to get me through the type of anxiety that you get with taking medication and so once that settled down I didn’t feel that I needed it anymore….”* **pBZD_07**  *“Well there were other things… it wasn’t just the seizure. It was part of the catalyst like for a couple of years I always knew I’d give them up eventually because I knew people that had been on them 30-40 years and I knew that wouldn’t be me do you know what I mean? I wouldn’t let that happen to me you know.”* **pBZD_06**  *“I suppose if it hadn't worked out, I could’ve gone come back to [the GP] and said, ‘Well, it's not working out and maybe I need to go back on them for another while’.”* **pBZD_02** |
| Environmental context and resource | - Lack of availability/access to relevant information and resources regarding BZRA discontinuation - Negative information regarding BZRA discontinuation process posted on online fora - Lack of support from healthcare professionals regarding discontinuation of BZRA use - Repeat BZRA prescribing system | - Availability/access to relevant information and resources regarding BZRA discontinuation - Support from healthcare professionals regarding discontinuation of BZRA use - Engagement with resources and supports for discontinuing BZRA use (e.g. counselling, sleep apps) - Pharmacological support to augment dosage reduction process (e.g. antidepressants) | *“I didn't find that there wasn't a single resource, maybe it is out there now, but I found at the time, there wasn’t a kind of a document that almost my GP could hand me. Here's a map to follow for the next 28 days to see how you get on”* **pBZD_02**  *“I remember looking up forums and looking up videos of people that had given them up and honestly there was nothing positive like. It scared me so much I remember watching one video and I got a panic attack”* **pBZD_12**  *“It was a case then that I wanted to get the prescription refilled, I called to the receptionist, I didn’t see the doctor. I went in and picked up a script”* **pBZD_05**  *“…. I was seeing a counsellor/psychiatrist at the time and she was such a great help. And she really understood the medication, and she was really helpful. And honestly, without her, I don’t know what I would have done.”* **pBZD_12**  *“They actually have specific mindfulness apps for people who can't sleep. I was going to say I didn’t find it hugely useful. The other thing I used actually were white noise apps. White noise apps I found were quite useful.”* **pBZD_02**  *“I was very lucky to have the psychologist and I think there is a huge gap in Ireland with that type of support that needs to be offered to everybody.”* **pBZD_08** |
| Social influences | - Lack of input from healthcare professionals regarding BZRA use/discontinuation - Negative nature of material posted online by online forum users regarding BZRA discontinuation | - Advice and support from healthcare professionals regarding discontinuation of BZRA use - Support from family and friends regarding discontinuation of BZRA use | *“The only person who intervened in any way was my pharmacist who expressed concerns that I was taking them for so long”* **pBZD_13**  *“No, I never felt like the GP had a strong feeling, like the GP wasn’t strongly pushing me to take it or pulling me to come off.”* **pBZD_09**  *“I would have read on the internet… the forums saying about how terrible it was and that you are better staying on them than having the withdrawal effects”* **pBZD_12**  *“I would attribute my success to coming off them with my GP. I had a very, very good relationship with her and she was extremely supportive”* **pBZD_10**  “I mean my family always encouraged me, my mother and father to give them up.” **pBZD_06** |
| Emotions | - Negative emotions (e.g. fear, worry) regarding discontinuation of BZRA use - Negative emotions (e.g. fear, worry) regarding potential for re-emergence of original symptoms^#^ | - None identified | *“It's a scary prospect. It is a really scary prospect. Not only are you dealing with all the stuff that you think is being suppressed but you are talking about dealing with withdrawals as well”* **pBZD_03**  *“And that [seizure] frightened the life out of me really if you know what I mean?* *So that spurred me on to give them up completely if you know what I mean”* **pBZD_06** |
| Behavioural regulation | - Limiting BZRA intake (i.e. using sparingly or omitting doses) | - Use of gradual dosage reduction - Use of pharmacological support to assist dosage reduction^#^ - Availability of reserve supply of BZRAs if needed^#^ - Use of resources and supports to augment dosage reduction process (e.g. sleep hygiene, mindfulness, counselling) | *“…she suggested that I take it on alternate nights and that was absolutely useless because the symptoms were so severe that that just meant that I didn’t sleep that night…so that just didn’t work at all...”* **pBZD_08**  *“I just did it. I just went from 20 down to 15, down to 10, down to five, like over about a period of six months. I just completely did it. I didn’t stop and then start again. I didn’t come down to 15 and go back up to 20.”* ***pBZD_01***  *“…the fact that I do have [zopiclone] if things get very, very, rough and I am having a really bad night or whatever I can take that and that does help me”* **pBZD_11**  “I started doing a bit of meditating and things like that to still the mind …” **pBZD_03**  “I always tried to see the positive in it and I used to tell myself I can do this and I can handle this…” **pBZD_13** |

^#^ Denotes barriers and facilitators that were unique to each patient cohort
